# Characterization of rhizome transcriptome and identification of a rhizomatous ER body in the clonal plant *Cardamine leucantha*

**DOI:** 10.1038/s41598-020-69941-9

**Published:** 2020-08-06

**Authors:** Kiwako S. Araki, Atsushi J. Nagano, Ryohei Thomas Nakano, Tatsuya Kitazume, Katsushi Yamaguchi, Ikuko Hara-Nishimura, Shuji Shigenobu, Hiroshi Kudoh

**Affiliations:** 1 Center for Ecological Research, Kyoto University, Otsu, Japan; 2 Department of Botany, Graduate School of Science, Kyoto, Japan; 3 National Institute for Basic Biology, Okazaki, Japan; 4 Present Address: Faculty of Life Sciences, Ritsumeikan University, Kusatsu, Japan; 5 Present Address: Faculty of Agriculture, Ryukoku University, Otsu, Japan; 6 Present Address: Department of Plant Microbe Interactions, Max Planck Institute for Plant Breeding Research, Cologne, Germany; 7 Present Address: Faculty of Science and Engineering, Konan University, Kobe, Japan

**Keywords:** Plant sciences, Plant ecology, Ecology, Molecular ecology, Transcriptomics

## Abstract

The rhizome is a plant organ that develops from a shoot apical meristem but penetrates into belowground environments. To characterize the gene expression profile of rhizomes, we compared the rhizome transcriptome with those of the leaves, shoots and roots of a rhizomatous Brassicaceae plant, *Cardamine leucantha.* Overall, rhizome transcriptomes were characterized by the absence of genes that show rhizome-specific expression and expression profiles intermediate between those of shoots and roots. Our results suggest that both endogenous developmental factors and external environmental factors are important for controlling the rhizome transcriptome. Genes that showed relatively high expression in the rhizome compared to shoots and roots included those related to belowground defense, control of reactive oxygen species and cell elongation under dark conditions. A comparison of transcriptomes further allowed us to identify the presence of an ER body, a defense-related belowground organelle, in epidermal cells of the *C. leucantha* rhizome, which is the first report of ER bodies in rhizome tissue.

## Introduction

Terrestrial plants spread their organs into two distinct spaces, i.e. above- and belowground. At the boundaries between above- and belowground spaces, physical conditions such as light, temperature and moisture change drastically. Furthermore, the presence of soil and litter forms the ground layer and provides specific biotic environments and plants are exposed to interactions with specific fauna and flora above- and belowground^1^. A plant therefore experiences two distinct environments composed of complex factors that interact physiologically and biologically^2,3^.

During the initial stage of plant growth after germination, two sets of organs, shoot and root systems, grow into the opposite directions to penetrate into aerial and soil spaces, respectively. Their growth and morphogenesis are distinctly determined by the shoot and root meristems. Both meristems contain stem cells whose identities and activities are regulated by intrinsic and environmental signals^4^. A shoot apical meristem produces leaves, stems and flowers and a root meristem primarily produces root.

Some groups of plants produce underground horizontal stems, i.e. rhizomes^5,6^. A rhizome originates from a shoot meristem and horizontally elongates underground through the expansion of internodes. Rhizomatous plants therefore provide an opportunity to compare above- and belowground shoots derived from the same type of meristem but that are exposed to contrasting environments. Leaves on rhizome nodes often become scale-like or rudimentary and stem internodes may elongate extensively^6^. As rhisomes function in energy storage, vegetative propagation and clonal spread,they are associated with plant productivity, competitiveness and invasiveness^7,8^. Thus, the development of rhizomes and the controls that regulate this process have been extensively studied in rhizomatous crops and wild plants^7,9,10^.

A comparison of transcriptomes between organs/tissues at the different developmental stages resulted in the detection of contrasting gene expression patterns^10–12^. Furthermore, unique genes expressed in particular organs/tissues have been identified^7,13^. Transcriptomic studies of rhizomes have been conducted in species such as *Sorghum* sp., *Phragmites australis* and *Oryza longistaminata*^7,9,10,14^. These studies have identified genes specifying rhizomes by comparing gene expression between rhizome tissues, e.g. tips and internodes^7,9,10,15^, between rhizomes and above-ground shoots or leaves^16–18^ and between rhizomes, roots and above-ground organs^16,19,20^.

A comparison of the transcriptomes of shoots and rhizomes derived from the same meristem but exposed to different environments under natural conditions provides concise information about the developmentally controlled robustness and plastic responses of plant shoot transcriptomes to the environment. Although developmental identity is considered to be the strongest determinant of transcriptomes, gene expression should also be largely determined by the environment surrounding an organ^21,22^. Differences in gene expression between rhizomes and shoots should therefore partly reflect the difference in physical and biological environments above and below ground. Furthermore, comparative transcriptomic analysis is expected to identify whether a rhizome has a specific system that is known to be either shoot or root specific. For example, the ER (endoplasmic reticulum) body is a log-shaped organelle constitutively present in the root and hypocotyl of *Arabidopsis thaliana* and related plants (predominantly in the Brassicaceae family) and contains a large amount of proteins with myrosinase (β-thioglucosidase) activity^23,24^, which is crucial for the production of key defense substances of Brassicaceae, i.e. glucosinolates in response to attack by herbivores and microbial pathogens. Thus, it has been proposed that ER bodies constitute the defense machineries of the roots, hypocotyls and cotyledons of *A. thaliana*^25,26^. Although the Brassicaceae family contains many rhizomatous species, it remains unclear whether ER bodies are present in rhizomes, one of the major belowground structures of plants.

**Figure 1:**
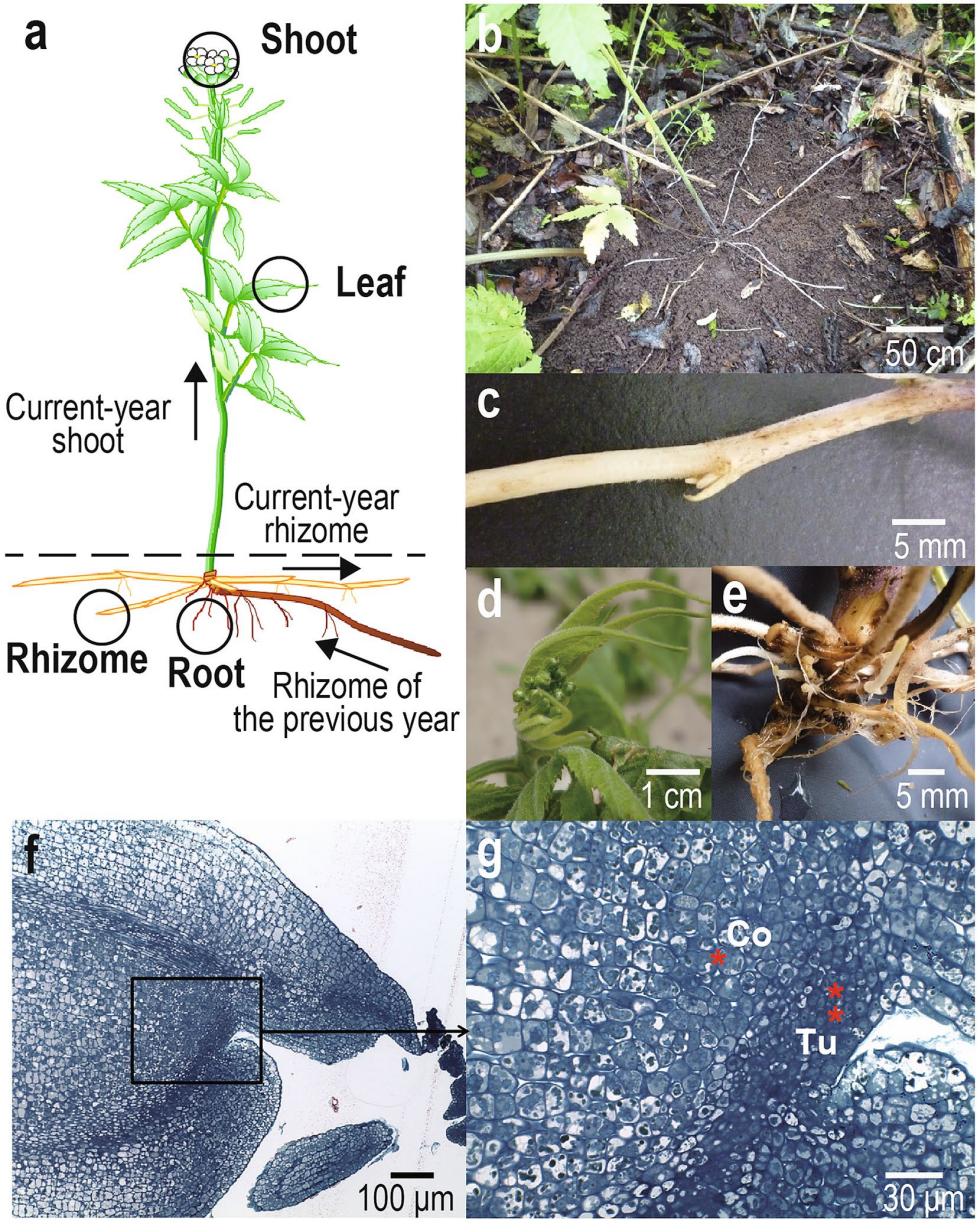
An illustration of four tissues (circles) collected from a current-year flowering shoot of a clonal rhizomatous plant, *Cardamine leucantha* (**a**), photographs of elongating belowground rhizomes (**b**), magnified view of a rhizome (**c**), floral bud and young leaves (**d**), rhizome tip and root (**e**) and tissue sections of a rhizome apex including its meristem (**f**,**g**). In (**a**), arrows indicate the direction of elongation of the current-year shoot, current-year rhizome and rhizome of the previous year and the dashed line represents the above- and belowground boundary. In (**f**) and (**g**), bars indicate scales and * and ** represent the corpus (Co) and tunica (Tu), respectively.

In this study, we aim to characterize the rhizome transcriptome of *Cardamine leucantha* (Brassicaceae) under natural conditions. The species is a clonal herbaceous plant that grows on deciduous forest floors and along forest margins. An aerial shoot elongates aboveground in spring from the tip of a rhizome that is produced in the previous year. The sequential expansion of leaves from aboveground shoots is followed by the production of flowers and the growth of the aboveground shoots stops with flowering and fruiting (Fig. 1a). At the same time, *C. leucantha* produces belowground, creeping, stoloniferous rhizomes at the base of the growing shoot (Fig. 1a). According to the morphological definition^6^^,^ the *C. leucantha* rhizome is classified as a secondary rhizome, originating from the lateral meristem of the main axis that forms the aboveground shoot. The developmental patterns of *C. leucantha* allowed us to simultaneously harvest four distinct tissues/organs, i.e. aboveground shoot, leaf, rhizome (belowground, derived from the shoot meristem) and root (belowground, derived from the root meristem), growing under identical climate conditions in the spring. In particular, we aimed to characterize the rhizome by comparing its transcriptome with other representative tissues. We specifically asked the following questions: (1) How similar is the transcriptome of the rhizome to that of the aboveground shoot and does their similarity reflect a shared developmental origin? (2) How similar is the transcriptome of the rhizome to that of the root and does their similarity reflect their shared belowground environment? (3) Are there any genes that are specifically expressed only in rhizomes?

## Results and discussion

### Gross morphological and anatomical observations of rhizomes

We conducted observations and sampling in a natural population of *C. leucantha* at a site in Rikubetsu, Hokkaido, Japan (43°27′ N, 143°46′ E, 251 m a.s.l.), located within a cool-temperate deciduous forest along the Toshibetsu River. The forest was dominated by *Salix sachalinensis* and contained *Fraxinus mandshurica, Quercus crispula* and *Ulmus davidiana* as common tree species. At this site, *C. leucantha* ramets elongated to form 30- to 60-cm upright stems and produced inflorescences with insect-pollinated white flowers in June. At the same time, one or more rhizomes started to elongate from the lateral shoot meristem at the basal part of the flowering shoot (Fig. 1b). A stoloniferous rhizome grew horizontally by elongating internodes (Fig. 1c), which then fully expanded by the end of the summer (23 cm on average, up to 65 cm in our observation). During the winter, the rhizome remained beneath the ground surface, when leaves and inflorescences had already formed at its tip. These shoot tissues appeared aboveground in the next growing season (Fig. 1d) and new rhizomes were observed at the base of the new shoot (Fig. 1e). We observed median longitudinal sections of tips of young rhizomes with toluidine blue stain under a microscope (Fig. 1f–g). The rhizome apex showed a typical shoot apical meristem structure with tunica/corpus organization (Fig. 1g), consistent with its developmental origin as a shoot apical meristem.

### Sequencing summary

We then collected RNA samples from *C. leucantha* plants at the study site in Rikubetsu to obtain the transcriptomic landscape of the *C. leucantha* tissues under natural biotic and abiotic conditions. We first collected RNA samples from four tissue types, i.e. a rhizome apex, a flowering shoot apex, a root apex and a leaf, at five time points from May 2011 to February 2012 for de novo assembly of the reference sequence with a high gene coverage. The cDNA sequencing data of these samples, obtained using the 454 Titanium platform (Roche, Basel, Switzerland), contained 1.5 M reads with an average length of 432 bp. In the final assembly using Newbler (454 Life Sciences; version 2.6), 27,834 isotigs (transcripts) were obtained with an average length of 1,386 bp and an N50 of 1,618 bp. These isotigs were then queried using the basal local alignment search tool (BLAST) Blastx (version 2.2.26) against *Arabidopsis thaliana* (TAIR10) protein data. 26,035 sequences (93.5%) successfully matched the database sequences with an e-value ≤ 1e−10. All 27,834 isotigs were used as the reference genes for the subsequent transcriptomic analysis. For transcriptomic resequencing, four issue types described above were collected from two plants in the flowering stage at noon on 31 May 2012 (Fig. 1a) and were subjected to RNA-Seq analysis. Hereafter, we refer to these two samples as plant A and plant B, respectively. Samples from the four tissues of plants A and B are referred to as rhizomes A and B, shoots A and B, leaves A and B, and roots A and B, respectively. These tissue samples were analysed using an Illumina HiSeq 2000 (Illumina, San Diego, CA, USA), using 2 × 101-bp paired-end sequencing, with one lane for two plants.

As a result of RNA-Seq, 10.9 M, 19.4 M, 17.7 M and 12.4 M reads in plant A and 21.8 M, 16.6 M, 15.2 M and 20.1 M reads in plant B were obtained for the rhizome, shoot, root and leaves, respectively. RNA-Seq data were mapped to the reference genes using Bowtie (version 0.12.8). For all samples, 65–80% of total reads were successfully mapped except for roots A and B (42% and 51%, respectively). The lower mapping rates in roots were partly due to the contamination of other organisms such as fungi, bacteria and virus, probably because the root samples were collected form natural forest soils.

### Overall characterization of the rhizome transcriptome

**Figure 2:**
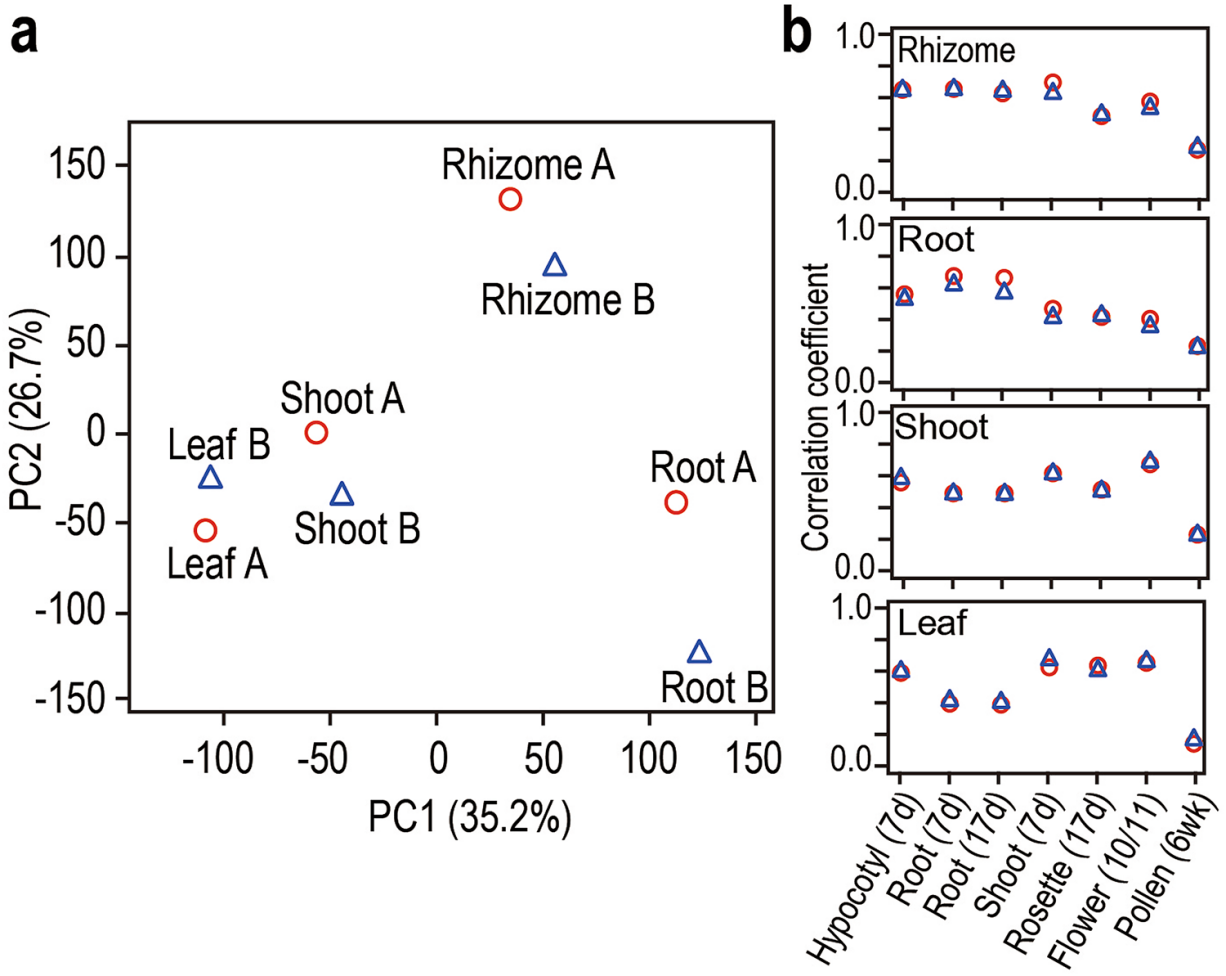
Principal component analysis (PCA) of transcriptome data of four tissues, i.e. rhizomes, shoots, roots and leaves of *Cardamine leucantha* (**a**). Spearman rank correlations between the *C. leucantha* transcriptome and *Arabidopsis thaliana* microarray (AtGenExpress) data for seven different tissues and developmental stages (**b**). In (**a**,**b**), red circles and blue triangles correspond to plants A and B, respectively. Shoot: vegetative_shoot_apex_7d, flower: flower_stage 10/11.

To compare the overall gene expression patterns of rhizomes, shoots, roots and leaves, PCA (principal component analysis) was conducted. The first and second axes explained 35.2% and 26.7% of the total variance, respectively (Fig. 2a). The patterns of plants A and B were consistent in which the rhizome, shoot and root samples were well separated. Shoot and leaf samples were closely located according to both the first and second axes. Along the first axis, rhizomes were located between shoot and root samples (Fig. 2a). Thus, the overall gene expression patterns of the rhizomes shared characteristics with those of both shoots and roots to some extent. Along the second axis, the scores of rhizomes deviated from those of the other tissues. This indicated that the expression of genes explained by the second axis was unique to the rhizomes.

The transcriptomes of the four tissues of *C. leucantha* were compared with microarray data from representative tissues of *A. thaliana*, i.e. hypocotyl, roots (7 days and 17 days old), vegetative shoot apex, rosette leaf, flower stage and pollen (AtGenExpress). The similarities of transcriptomes were evaluated by Spearman’s rank correlation coefficients (Fig. 2b). For both plants A and B, the expression patterns of the rhizome of *C. leucantha* showed a relatively high correlation with those of the roots (7 and 17 days old) and hypocotyl and shoot apex of *A. thaliana* (Fig. 2b). The results support the idea that the rhizome shares characteristics with the shoot and root. The gene expression patterns of the shoot, root and leaf samples of *C. leucantha* were highly correlated with the corresponding tissues of *A. thaliana*. The shoot transcriptome of *C. leucantha* showed the highest similarity with that of *A. thaliana* flower likely because the shoot samples of *C. leucantha* in spring contained young flowering buds (Fig. 2b).

### Transcript clustering based on tissue-dependent expression patterns

**Figure 3:**
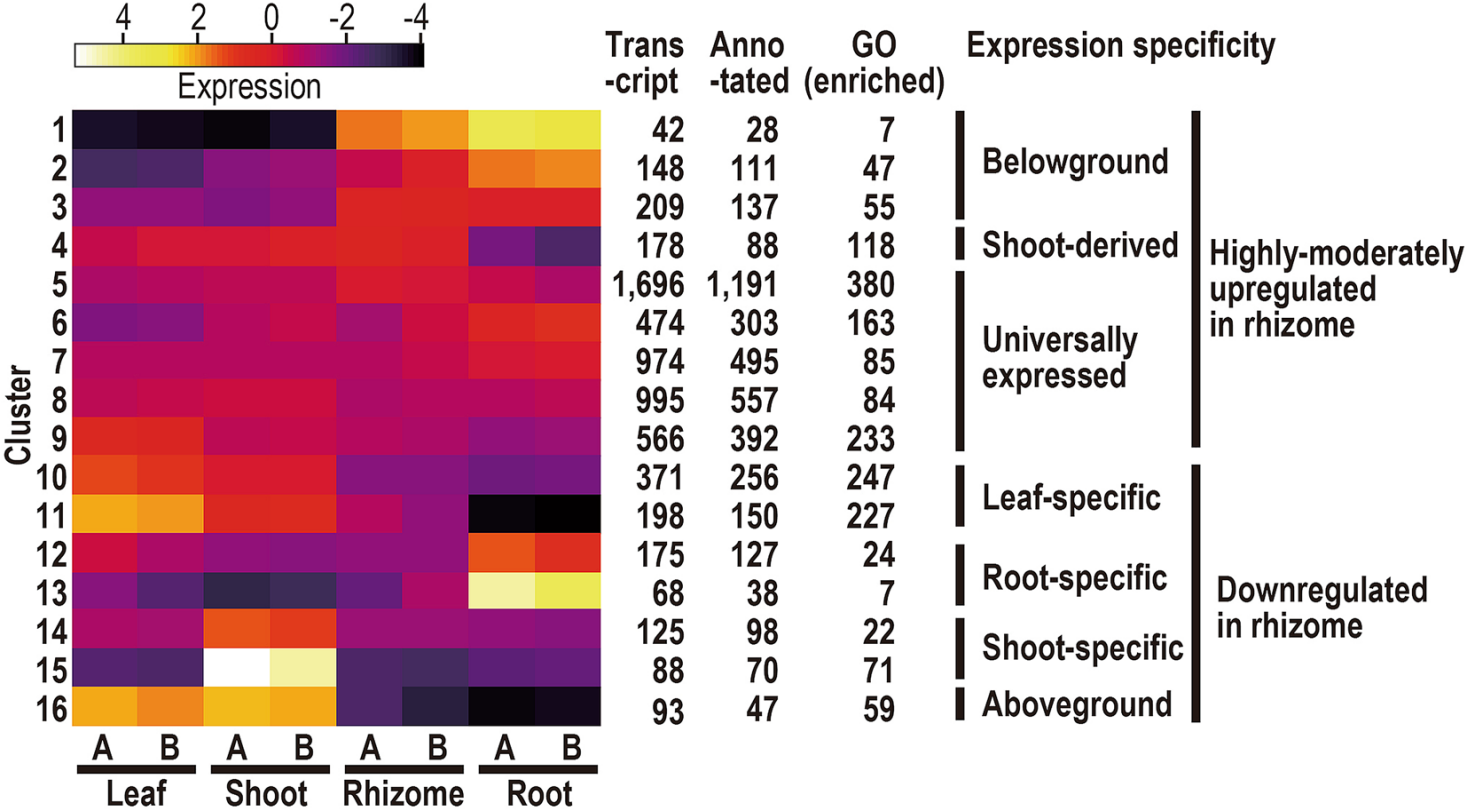
A heatmap of 16 k-mean clusters classified by relative expression patterns in four tissues, i.e. leaf, shoot, rhizome and root, of *Cardamine leucantha* plants A and B. Numbers of transcripts, annotated transcripts and enriched gene ontologies (GOs) for each cluster are also listed. Expression specificity based on the expression pattern is indicated by vertical bars.

K-mean clustering resulted in classification of all expressed genes into 16 clusters based on the gene expression patterns across the four tissues from plants A and B (Fig. 3). The number of transcripts included in each cluster ranged from 42 (cluster 1) to 1,696 (cluster 5). Significantly enriched gene ontologies (GOs) within clusters ranged from seven in clusters 1 and 13 to 380 in cluster 5 (*P* < 0.05, Supplementary Table S1).

Remarkably, none of the clusters exhibited rhizome-specific expression patterns, whereas several clusters of genes were expressed in shoot- and root-specific manners (Fig. 3). Transcripts that showed relatively high expression in rhizomes were grouped in clusters 1–9 which were also highly expressed in the roots (clusters 1–3) or in the aerial tissues (cluster 4, Fig. 3). Cluster 1 was significantly enriched in cell wall-related GOs (GO:0015928, fucosidase activity; GO:0005199, structural constituent of cell wall) and symbiosis-related GOs (GO:0009610, response to symbiotic fungus; GO:0009608, response to symbiont, Supplementary Table S1). Clusters 5–9 were universally expressed across the four tissues. These observations were consistent with the intermediate locations of the rhizome transcriptome relative to the root and aboveground transcriptomes in the PCA plot. In contrast, the transcriptomic profiles of the other three tissues were characterized by the clusters specific to each tissue. Clusters 10 and 11, 12 and 13, and 14 and 15 were specific to leaf, root and shoot tissues, respectively and cluster 16, which was highly enriched by cuticle-wax-related GOs, represented transcripts that were specific to aboveground tissues (Fig. 3).

### Representative transcripts of the rhizome as a belowground structure

Clusters 1–3 represented transcripts whose expressions were higher in the rhizome and root than in the leaf and shoot. In particular, genes in cluster 1 were exclusively expressed in rhizomes and roots and were nearly absent from the aboveground transcriptomes (Fig. 3). Representative transcripts in cluster 1 contained those annotated to *A. thaliana* genes related to belowground defense and the control of reactive oxygen species (ROS), which suggested a functional aspect of the rhizome as a belowground organ. The top seven transcripts with the highest average rhizome expressions in cluster 1 (Table 1) were annotated to *Pathogenesis-related protein 4* (*PR-4*, AT3G04720), *Light-dependent Short Hypocotyls 10* (*LSH10*, AT2G42610), *Peroxidase 37* (*AtPrx37*, AT4G08770), *GDSL-like Lipase 23* (*GLL23*, AT1G54010), *Peroxidase 39* (*AtPrx39*, AT4G11290), *Methionine Sulfoxide Reductase B9* (*MSRB9*, AT4G21850) and *β-glucosidase 23* (*PYK10 /BGLU23*, AT3G09260). PR-4 is involved in JA (Jasmonic Acid)-dependent defenses and is upregulated by treatment with necrotrophic fungi^27^ and rhizobacteria^28^. A homolog of *LSH10* in potato (*Solanum tuberosum* L.) has been reported to show very high transcript levels in stolons (rhizomes) and young tubers^29^. *AtPrx37* encodes a peroxidase superfamily protein that has been reported to be expressed in the roots of *A. thaliana*, as well as in the basal parts of flowering stalks and mature rosette leaves^30^. Furthermore, *AtPrx37* was reported to be up-regulated in *A. thaliana* roots in response to increasing ROS concentration under nitrogen, phosphorus, and potassium deficiency and is responsible for mineral uptake^31^. *AtPrx39* was also reported to be involved in the control of the balance between distinct classes of ROS in the roots of *A. thaliana,* thereby regulating root meristem homeostasis^32^. *MSRB9* has been reported to be prevalently expressed in *A. thaliana* roots and is involved in tolerance to the accumulation of ROS^33^. Overall, these findings suggest that the rhizome defense machinery possesses a belowground characteristic.

**Table 1:** Representative transcripts of *Cardamine leucantha* that showed specific belowground expression.

Cl. No	*Cardamine leucantha* transcripts	Expression (Log_2_ FPKM)								AGI code	Short description
		Leaf		Shoot		Rhizome		Root			
		A	B	A	B	A	B	A	B		
1	isotig23034	1.26	0.28	− 0.59	0.41	7.61	7.20	8.36	4.37	AT3G04720	Pathogenesis-related protein 4 (PR-4)
1	isotig20431	− 0.33	− 0.16	–	− 1.46	7.34	7.44	4.72	5.05	AT2G42610	Light-dependent Short Hypocotyls 10 (LSH10)
1	isotig06471	1.52	− 0.46	0.76	− 0.08	7.04	7.61	8.15	3.66	AT4G08770	Peroxidase 37 (AtPrx37 /Per37)
1	isotig17255	− 3.59	− 1.28	–	–	6.61	6.74	7.68	6.75	AT1G54010	GDSL-like Lipase 23 (GLL23)
1	isotig19263	− 0.09	− 4.64	–	–	6.72	6.45	3.88	3.48	AT4G11290	Peroxidase 39 (AtPrx39 /Per39)
1	isotig11418	− 4.23	-	–	–	6.79	5.67	5.84	3.62	AT4G21850	Methionine Sulfoxide Reductase B9 (MSRB9)
1	isotig03131	–	–	− 1.88	0.66	5.91	6.07	8.71	8.52	AT3G09260	β-Glucosidase 23 (BGLU23/PYK10)

The top seven transcripts with the highest average rhizome expressions among annotated genes in cluster 1 are shown. Cluster number in k-mean analyses (Cl. no.), transcript ID in *C. leucantha* (isotig number denoted by Newbler), gene expression (Log_2_ FPKM) in leaf, shoot, rhizome and root of plants A (A) and plant B (B), and AGI code and short description of corresponding annotated genes in the *Arabidopsis thaliana* database (TAIR10 protein sequences) are listed.

Cluster 16, which contained genes that were specifically expressed in aboveground tissues but not in roots and rhizomes, also characterized the transcriptome of the rhizome as a belowground organ (Fig. 3). For instance, *CER1* in this cluster encodes an enzyme that converts leaf/stem wax C30 aldehydes to C29 alkanes^34,35^ suggesting that the composition of cuticular wax in rhizomes may be different from that of stems and leaves.

### Representative transcripts of the rhizome as a shoot-derived structure

In contrast to the aforementioned clusters, transcripts in cluster 4 were highly expressed in rhizomes as well as in shoots and leaves, but only moderately in roots (Fig. 3 and Table 2). The top seven transcripts with the highest average rhizome expressions among annotated genes in cluster 4 (Table 2) were *Lipid Transfer Protein 2* (*LTP2*, AT2G38530), *β-glucosidase 18* (*BGLU18*, AT1G52400), a cell wall protein (AT2G10940), *Epithiospecifier Protein* (*ESP*, AT1G54040), *Glutathione Peroxidase 3* (*GPX 3*, AT2G43350), *Cell Wall-Plasma Membrane Linker Protein* (*CWLP*, AT3G22120) and *GAST1 Protein homolog 4* (GASA4, AT5G15230). The *LTP2* transcript in *A. thaliana* was reported to be highly accumulated in the epidermal cells of the hypocotyl and cotyledons in dark-grown seedlings^36^. In *A. thaliana*, ESP was found to be consistently present in the epidermal cells of all aerial parts^37^ and encodes a myrosinase cofactor, which is necessary to drive the myrosinase-catalyzed hydrolysis of glucosinolates and prompts terminal production of nitriles and epithionitriles in *Brassica* and *Arabidopsis*^38^. Collectively, high levels of transcripts homologous to these genes in *C. leucantha* rhizomes likely represent a shoot-derived tissue, particularly one growing under dark conditions.

**Table 2:** Representative transcripts of *Cardamine leucantha* that showed shoot-derived tissue-specific expression.

Cl. no	*Cardamine leucantha* transcripts	Expression (Log_2_ FPKM)								AGI code	Short description
		Leaf		Shoot		Rhizome		Root			
		A	B	A	B	A	B	A	B		
4	isotig25586	9.15	9.60	9.21	8.62	9.30	9.44	8.36	5.81	AT2G38530	Lipid transfer protein 2 (LTP2)
4	isotig00469	7.57	7.99	8.46	9.40	9.22	8.76	5.71	4.60	AT1G52400	β-Glucosidase 18 (BGLU18)
4	isotig03397	9.40	9.19	7.80	6.97	8.84	8.88	5.47	4.26	AT2G10940	Bifunctional inhibitor/lipid-transfer protein/seed storage 2S albumin superfamily protein
4	isotig14602	8.72	9.10	8.23	8.54	8.87	8.10	5.16	6.05	AT1G54040	Epithiospecifier protein (ESP)
4	isotig03283	8.41	8.39	8.15	7.83	8.22	8.48	5.27	3.97	AT2G43350	Glutathione peroxidase 3 (GPX3)
4	isotig03286	8.35	8.35	7.92	7.73	8.17	8.52	5.09	3.89	AT3G22120	Cell wall-plasma membrane linker protein (CWLP)
4	isotig25402	7.98	7.48	7.95	7.10	8.26	8.07	5.58	3.93	AT5G15230	GAST1 protein homolog 4 (GASA4)

The top seven transcripts with the highest average rhizome expressions among annotated genes in cluster 4 are shown. Cluster number in k-mean analyses (Cl. no.), transcript ID in *C. leucantha* (isotig number denoted by Newbler), gene expression (Log_2_ FPKM) in leaf, shoot, rhizome and root of plants A (A) and plant B (B), and AGI code and short description of corresponding annotated genes in the *Arabidopsis thaliana* database (TAIR10 protein sequences) are listed.

### Transcripts with high expression in the rhizome relative to other tissues

Because there was no k-mean cluster that showed strong rhizome-specificity in its expression, we compared the expression between four tissues for each transcript. We found that 394 transcripts annotated to 172 *A. thaliana* genes showed the maximum expression level in rhizomes, with twofold higher expression compared to the tissue with the second highest expression level (Supplementary Table S2). The top three genes in the difference of expression compared with the tissue with the second highest expression level showed 27-, 14- and 12-fold differences in the FPKM value and 23 transcripts showed more than fivefold differences (listed as the Log_2_ FPKM difference in Table 3, Supplementary Table S2). The top one was annotated to a gene (AT4G22485) encoding a lipid-transfer protein whose function has not been addressed (Table 3). The transcript with the second highest expression level was annotated to a gene coding a cell-wall loosing protein, *Expansin 3* (*EXPA3*, AT2G37640), that promotes cell expansion in the roots of *A. thaliana*^39,40^. Furthermore, two additional transcripts within the top 10 were annotated to *A. thaliana TUB1* (β-tubulin; AT1G75780) and *ARR3* (type-A response regulator; AT1G59940), both of which are potentially related to root/hypocotyl elongation. *TUB1* expression has been reported to be high in etiolated seedlings of *A. thaliana* and is suppressed by light^41^. *ARR3* has been reported to be constitutively expressed in the vascular tissue of both shoots and roots and is induced by cytokinin in root tissues^42^. These observations are consistent with the extensive elongation of *C. leucantha* rhizomes under dark belowground conditions.

**Table 3:** Ten annotated transcripts of *Cardamine leucantha* that showed the highest expression in the rhizome relative to the tissue with the second highest expression.

Cl. no	*Cardamine leucantha* transcripts	Expression (Log_2_ FPKM)								Tissue with 2nd highest FPKM	Rhizome – 2nd (Log_2_ FPKM)	AGI code	Short description
		Leaf		Shoot		Rhizome		Root					
		A	B	A	B	A	B	A	B				
4	isotig20852	0.43	2.06	–	1.90	6.47	5.54	–	–	Leaf	4.76	AT4G22485	Bifunctional inhibitor/lipid-transfer protein/seed storage 2S albumin superfamily protein
5	isotig18882	2.75	2.46	2.34	1.77	6.59	6.30	2.47	1.45	Leaf	3.84	AT2G37640	Cell-wall loosing protein, Expansin 3 (EXP3)
3	isotig23165	1.67	1.38	–	2.50	4.28	6.72	2.03	1.81	Root	3.59	AT4G17470	alpha/beta-Hydrolases superfamily protein
3	isotig26188	− 0.58	–	0.77	0.39	4.44	5.24	1.90	0.75	Root	3.52	AT4G10265	Wound-responsive family protein
5	isotig05949	1.81	0.26	2.95	1.68	5.85	5.03	1.93	0.93	Shoot	3.12	AT2G44770	ELMO/CED-12 family protein
5	isotig05947	1.84	0.70	3.15	1.98	6.00	5.28	2.14	1.06	Shoot	3.08	AT3G60260	ELMO/CED-12 family protein
3	isotig27512	− 1.04	− 1.88	− 1.31	–	4.99	5.42	2.92	1.56	Root	2.97	AT4G24275	Uncharacterized protein
1	isotig19263	− 0.09	− 4.64	–	–	6.72	6.45	3.88	3.48	Root	2.91	AT4G11290	Peroxidase 39 (AtPrx39 /Per39)
3	isotig15192	3.70	3.70	3.83	3.98	7.11	7.02	4.25	4.23	Root	2.82	AT1G75780	Tubulin β-1 chain (TUB1)
3	isotig20412	1.69	1.59	1.05	0.93	4.96	5.59	2.94	2.18	Root	2.72	AT1G59940	Cytokinin inducible Type A response regulator (ARR3)

Cluster number in k-mean analyses (Cl. no.), transcript ID in *C. leucantha* (isotig number denoted by Newbler), gene expression (Log_2_ FPKM) in the leaf, shoot, rhizome and root of plants A (A) and B (B), tissue with the 2nd highest FPKM value, FPKM difference between the rhizome and the tissue with the 2nd-highest expression (Log_2_ FPKM), and AGI code and short description of corresponding annotated genes in the *Arabidopsis thaliana* database (TAIR10 protein sequences) are listed.

### Enrichment of ER body-related genes and identification of ER bodies in the rhizome

**Figure 4:**
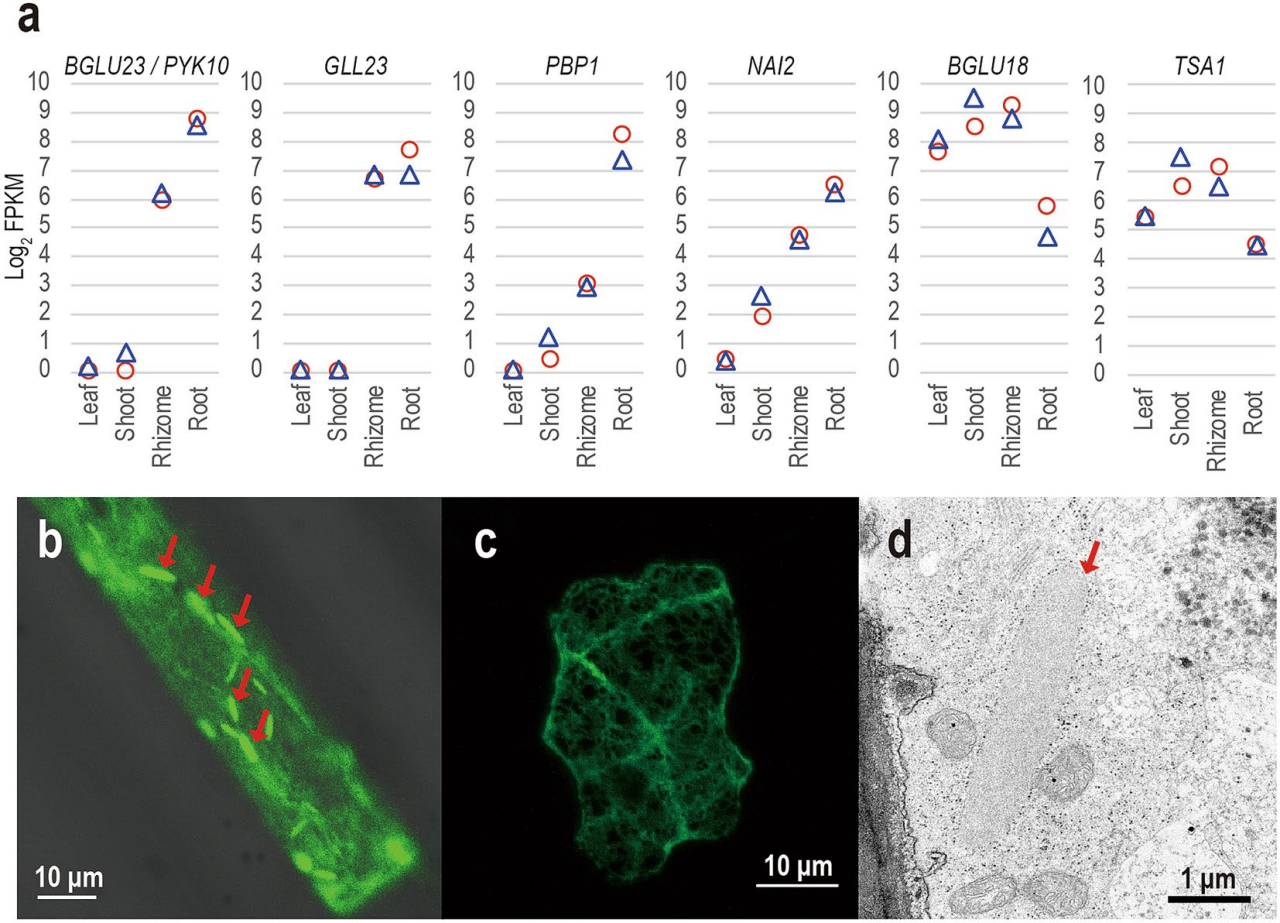
Expression (log_2_ FPKM) of six ER-body-related genes across four tissues (**a**), fluorescent microscope images of epidermal cells in the rhizome (**b**) and mature leaf (**c**), and a transmission electron microscope (TEM) image of an epidermal cell in the rhizome (**d**) of *C. leucantha*. In (**a**), red and blue circles represent plants A and B, respectively. In (**b**,**c**), GFP targeted to ER was transiently expressed. In (**b**,**d**), putative ER bodies are indicated by red arrows. Bars indicate ten micrometres in (**b**,**c**) and one micrometre in (**d**).

We noted that the transcripts annotated to the key genes for ER-body formation were highly expressed in the rhizome tissue of *C. leucantha* (Fig. 4a)*.* A gene homologous to *BGLU23/PYK10*, encoding the major component of the ER bodies, was highly expressed in the rhizomes and roots of *C. leucantha* but was absent in the leaves and shoots (Fig. 4a and Table 1). The homologs of genes encoding GDSL lipase-like protein 23 (*GLL23*, AT1G54010) and PYK10-binding protein1 (*PBP1*, AT3G16420) also showed high expression in both rhizomes and roots. In *A. thaliana*, these proteins have been reported to be members of the PYK10 complex^43,44^, which is a huge protein aggregate that facilitates the enzymatic activity of PYK10^45^. For transcripts homologous to the gene essential for ER body formation, *NAI2*, and its homolog *TSA1* (AT3G15950 and AT1G52410)^46,47^, the gene encoding the major component of leaf-type ER bodies^48^^,^ were also highly expressed in rhizomes of *C. leucantha* (Fig. 4a and Table 2). Furthermore, transcripts related to the biosynthesis of indole glucosinolate (*CYP79B2*, AT4G39950; *CYP83B1*, AT4G31500; *TSA1*, AT3G54640), which was proposed to be the *in planta* substrate of PYK10^24^^,^ were also enriched in the rhizome (Clusters 3 and 4,Supplementary Table S2).

Enrichment of expressions of ER body-related genes in the rhizome prompted us to test the presence of ER bodies in *C. leucantha* rhizomes. We visualized the ER in the epidermal cells of rhizomes and leaves by transiently expressing green fluorescent protein (GFP) fused with a signal peptide and an ER-retention signal (SP-GFP-HDEL). In the rhizomes, rod-shaped structures were detected in addition to the typical ER network (Fig. 4b). These were absent in the leaf epidermal cells (Fig. 4c). These structures were similar to the ER bodies in *A. thaliana* in both shape and size, suggesting that they correspond to the ER bodies of *C. leucantha*. This observation was further corroborated by the electron micrographs of rhizomes that delineated the presence of a spindle-shaped compartment with ribosomes on its cytosolic surface, which resembled the ER bodies in *A. thaliana*. Interestingly, we found amyloplasts in rhizome cells, i.e. organs containing starch grains in a plastid, but not chloroplasts (Fig. 4d), suggesting that the rhizome of *C. leucantha* serves as the energy storage organ^49–51^. Overall, these results clearly demonstrate that the rhizome of *C. leucantha* develops ER bodies similar to the roots of the other *Brassicaceae* plants. Notably, in *C. leucantha* rhizomes*,* the ER bodies appeared to contain proteins homologous to *PYK10* or to *BGLU18*, which are the major components of constitutive and facultative ER bodies, respectively. This might suggest that the *C. leucantha* rhizome ER bodies might represent both types of ER bodies, which is consistent with our comparative transcriptomic analysis that revealed the characteristic of rhizomes to be intermediate between the above- and belowground tissues.

### Reproducibility of gene expression patterns

To evaluate the reproducibility of the results of RNA-Seq, we conducted real-time qPCR experiments using the three independent sets of four tissues, i.e. rhizomes, shoots, roots and leaves, from three plants. We examined ten selected homologous genes, i.e. *LSH10* (isotig20431), *Prx37* (isotig06471), *GLL23* (isotig17255), *PYK10* (isotig03131), *BGLU18* (isotig00469), *PBP1* (isotig17838), *NAI2* (isotig07117), *TSA1* (isotig02612), *AGL9* (isotig04090) and *PHOT1* (isotig12495). Gene expressions were reproduced in all genes at least in the rank order across four tissues except for those of *PBP1* and *PHOT1* (Fig. S1).

Expressions of additional eight genes., i.e. *AGT* (isotig04516), *CA1* (isotig18828), *CER* (isotig00800), *ER* (isotig12468), *EXT19* (isotig00056), *MYB15* (isotig19642), *PSBX* (isotig27053) and *PUB23* (isotig08161), whose patterns were extremely different between leaf (aboveground part) and root (belowground one) were also checked by real-time qPCR. The expression patterns were mostly similar to those of RNA-Seq (Fig. S2).

## Conclusions

A comparison of the transcriptomes of rhizomes, shoots, roots and leaves in the rhizomatous plant *C. leucantha* revealed that the rhizome has a transcriptome characterized as an intermediate between the shoot and the root. Previous studies investigating rhizome transcriptomes have reported a clear difference in gene expression pattern between the rhizome and aerial shoots/leaves in bamboo^15^^,^*Oryza*^16^^,^*Sorghum*^7,52^ and *Atractylodes*^18^^,^ however, its relationship to the transcriptome of belowground organs such as roots remained unclear. We included root samples in our analyses and showed that the rhizome and the root strongly share transcriptomic patterns likely due to the shared belowground environments. The overall characteristic of the rhizome transcriptome is intermediate between those of the aboveground shoot and the belowground roots, suggesting that both endogenous developmental and external environmental factors are important in the regulation of the rhizome transcriptome. Comparisons between rhizomes, roots, leaves and shoots have been reported for clonal grass species such as *Oryza*^16,53^ and *Miscanthus*^19,20^. The reported lists of the genes that were highly expressed in the rhizomes in these studies did not correspond to that of this study, at least for the top twenty genes, probably reflecting distant phylogenetic relationships between monocots and dicots. The comparison of transcriptomes further allowed us to identify the presence of ER bodies, defense-related belowground organelles, in epidermal cells of *C. leucantha* rhizomes, which is the first report of an ER body in rhizome tissue. Our study suggested that the surrounding environment largely influenced organ identity at the gene expression level, but further studies are required to determine how much of the rhizome-specific expression patterns are constitutive and/or responsive to surrounding environments.

## Materials and methods

### Tissue sampling of plant species

*Cardamine leucantha* (Tausch) O. E. Schulz [Brassicaceae] is a diploid clonal herb (2n = 16). Because the genus *Cardamine* is closely related to a model species, *A. thaliana*^54,55^, we can take advantage of annotated genes and can estimate their functions using available information^55,56^. De novo assembly samples were collected from plants that were transplanted from the original Rikubetsu population to the garden of the Center for Ecological Research (CER), Kyoto University (34°58′ N, 135°57′ E, 152 m a.s.l.). Samples from the four tissue types, i.e. a rhizome apex, a flowering shoot apex, a root apex and a leaf, were collected 5 times in various seasons from May 2011 to February 2012 to maximize the number of genes included in the reference. For transcriptomic resequencing, six plants in the flowering stage were carefully dug up at noon on 31 May 2012 in Rikubetsu population, Hokkaido. The four tissue types described above were collected from each plant (Fig. 1a). Tissues were preserved in RNAlater (Life Technologies, Carlsbad, CA, USA) on site and were transported to the laboratory on ice. The samples were kept at − 80 °C until RNA extraction. Finally, one mixed RNA samples containing four tissues for de novo sequencing. Eight RNA samples of four tissues from two plants were subjected to RNA-Seq analysis based on the RNA quality.

### Sequence analysis

RNA-Seq samples were processed by HiSeq 2000 sequencing (Illumina) to obtain sequences of fragments derived from mRNA. From each sample, RNA was extracted using an RNeasy Mini Kit (QIAGEN, Hilden, Germany). The quality of extracted RNA was determined using an Agilent 2,100 Bioanalyzer (Agilent; Palo Alto, CA, USA). The RNA concentration of the extracts was measured by Qubit (Invitrogen; Thermo Fisher Scientific, Carlsbad, CA, USA) to determine the quantity of the samples used for library preparation. RNA-Seq libraries were prepared using the Illumina TruSeq RNA sample preparation kit (low-throughput protocol, Illumina) following the manufacturer’s instructions. For each sample, 0.1–0.5 μg of total RNA was used.

De novo assembly samples were processed by the 454 Titanium platform to obtain cDNA sequences of *C. leucantha*. These de novo assembled data were used to construct the references onto which the RNA-Seq data were mapped. Total RNA was collected from four tissues in the different seasons described above using RNeasy Mini Kit (QIAGEN) before being purified twice by Oligo (dT) 25 Dynabeads. All samples were pooled as a 290-ng mRNA sample to synthesize cDNA. The mixed tissue sample library was prepared using the GS Titanium Rapid Library Preparation Kit (Roche, Basel, Switzerland) following the manufacturer’s protocol and was then analysed using a 454 Titanium platform, with a single end in one plate.

### Data analysis

For both RNA-Seq data and de novo assembled data, sequences were checked for the quality and were trimmed using the FASTX toolkit. De novo assembly was conducted using the Newbler programme^57^. Assembled contigs were BLAST queried and annotated to the *A. thaliana* database (27,416 protein data points in TAIR 10) using Blastx. The top hit gene with an e-value <  = 1e−10, was treated as an ortholog. RNA-Seq reads were then mapped to the references and counted using Bowtie^58^. RNA-Seq reads were mapped to the references and the FPKM (fragments per kilobase of transcript per million fragments mapped) values were calculated. Because the chemicals used in Illumina sequencing differed between the first and second runs, the RNA-Seq data for plants (A and B) were analysed separately.

FPKM values were normalized by the quantile-spline method using the normalize qspline function in the affy package of R^59^. Log_2_-transformed values of the normalized FPKM plus 2^–5^ were used as expression values (Supplementary Data S1). Principal component analysis (PCA) was performed using the prcomp function in R to compare the overall gene expression patterns of the rhizome, shoot and root apexes and leaves. Genes whose expressions were higher than 0 in at least one sample were used in the PCA. We performed k-mean clustering using the k-mean function in R. The number of clusters was set to 16. Genes whose expressions were higher than 5 in at least one sample were used in the k-mean clustering. GO enrichment analyses were conducted for each cluster.

In order to characterize the transcriptomes of the four tissues of *C. leucantha*, especially that of the rhizome tip, the gene expression patterns were compared with previously reported transcriptome data from other species. First, comparisons were made with *A. thaliana* microarray data for seven selected tissues collected as part of the AtGenExpress project: the hypocotyl 7 days after germination (d.a.g.) (ATGE_2), the root at 7 d.a.g. (ATGE_3), the root at 17 d.a.g. (ATGE_9), the vegetative shoot apex at 7 d.a.g. (ATGE_6), the rosette leaf at 17 d.a.g. (ATGE_12), mature pollen 6 weeks after germination (ATGE_57) and the flower at stage 10/11 (ATGE_31)^60^. The Spearman’s rank correlation between each of *C. leucantha* transcriptome and each *A. thaliana* transcriptome was calculated. Putative orthologous genes in *A. thaliana* were used in the calculation.

### Real-time qPCR

To confirm the reproducibility of gene expression differences between four tissues in the RNA-Seq, quantitative real-time qPCR was performed on independent samples. Ten selected homologous genes, i.e. *LSH10*, *Prx37*, *GLL2*3, *PYK10*, *BGLU18*, *PBP1*, *NAI2*, *TSA1*, *AGL9* and *PHOT1* characterizing rhizome transcriptomes were relatively quantitated by *Actin2* (isotig15388) as a reference gene (Supplementary Table S3). Eight selected genes, i.e. *AGT*, *CA1*, *CER*, *ER*, *EXT19*, *MYB15*, *PSBX* and *PUB23* characterizing leaf or root transcriptomes were selected to confirm the reproducibility of gene expression of roots (Supplementary Table S3). Samples of four tissues (leaf, shoot, rhizome and root) or two tissues (leaf and root) from three plants were analyzed by real-time qPCR (Thermal Cycler Dice Real Time System III, TaKaRa, Shiga, Japan). Samples were collected in a population of Shiga, Japan (N35°15′, E136°21′, 261 m a.s.l.) in May 2013.

### Anatomical observation and subcellular structure of rhizome tips

In order to anatomically characterize the rhizome tips of *C. leucantha*, bright-field microscopy and transmission electron microscopy were used. The rhizome tip samples were collected in June when the rhizome elongated actively from the transplants. The samples were fixed with 2% paraformaldehyde (PFA) and 2% glutaraldehyde (GA) in 0.05 M cacodylate buffer (pH 7.4) at 4 °C overnight. After fixation, the samples were rinsed three times with 0.05 M cacodylate buffer for 30 min each, followed by post fixation with 2% osmium tetroxide (OsO4) in 0.05 M cacodylate buffer at 4 °C for 3 h. The rhizome tips were observed using a transmission electron microscope (JEM-1200 EX; JEOL Ltd., Tokyo, Japan) at an acceleration voltage of 80 kV. Digital images were taken using a CCD camera (VELETA, Olympus Soft Imaging Solutions GmbH, Münster, Germany).

To examine the presence or absence of ER bodies in leaves and rhizomes of *C. leucantha*, tissues were collected from plants transplanted in the CER garden (described above). The ER body is reported to be present in the roots and cotyledons and absent in the leaves and stems of *A. thaliana*^25^. A fluorescence producing gene, SP-GFP-HDEL, which is expected to localize GFP in the endoplasmic reticulum (ER) and ER-derived organelles^61,62^ was transiently introduced into the collected tissues by particle bombardment, as described previously^63^. The tissues were then incubated for 2 days in a 22 °C chamber under continuous light with 100% humidity. GFP-expressing cells were inspected under a confocal laser-scanning microscope (LSM780 META; Carl Zeiss, Oberkochen, Germany).

## Supplementary information

Supplementary file1

Supplementary file2

Supplementary file3

Supplementary file4

## Data Availability

The *C. leucantha* raw sequencing data are available from the DDBJ BioProject database (BioProject Accession number: PRJDB9665, BioSample Accession number: SAMD00217524) for sequencing by 454 Titanium and (BioProject Accession number: PRJDB9421, BioSample Accession number: SAMD00209866—SAMD00209873) for sequencing by Illumina HiSeq2000. Isotig data was deposited in the Dryad Digital Repository: 10.5061/dryad.r4xgxd28x.
