# Supplementary material for: Characterization of rhizome transcriptome and identification of a rhizomatous ER body in the clonal plant Cardamine leucantha
Source: Sci Rep. 2020 Aug 6;10:13291. doi: 10.1038/s41598-020-69941-9 (PMC7413523; doi:10.1038/s41598-020-69941-9)
Supplement: Supplementary file 4 — Supplementary file4 [file 41598_2020_69941_MOESM4_ESM.pdf]

**Characterization of rhizome transcriptome and identification of a rhizomatous ER body in the clonal plant *Cardamine leucantha***

Kiwako S. Araki, Atsushi J. Nagano, Ryohei Thomas Nakano, Tatsuya Kitazume, Katsushi Yamaguchi, Ikuko Hara-Nishimura, Shuji Shigenobu & Hiroshi Kudoh

**Supplementary Table S3. Primers for real-time qPCR of *Cardamine leucantha* .** Primers designed on 19 transcripts of *Cardamine leucantha* .

| Name | Sequence (5'-3') | Transcript of *Cardamine leucantha* | AGI code | Short description |
|---|---|---|---|---|
| *CleLSH10* _F | TTTGATCGGACGGCTGAGA | isotig20431 | AT2G42610.2 | Light-dependent Short Hypocotyls 10 (LSH10) |
| *CleLSH10* _R | GATTCGTTTCCGGAGATCCA | | | |
| *ClePrx37* _F | GCAGCCTTCATTTTGTCGATTA | isotig06471 | AT4G08770.1 | Peroxidase 37 (AtPrx37 /Per37) |
| *ClePrx37* _R | TGCATTTGGGAACGCAAGA | | | |
| *CleGLL23* _F | GAGTTTGCCGTTGGGATCA | isotig17255 | AT1G54010.1 | GDSL-like Lipase 23 (GLL23) |
| *CleGLL23* _R | ACGTCGCTCAAGGTTTTTGG | | | |
| *ClePYK10* _F | TCGAAGCACATGACCTTGCT | isotig03131 | AT3G09260.1 | β-Glucosidase 23 (BGLU23/PYK10) |
| *ClePYK10* _R | TCAAGCGCACGGTCAATG | | | |
| *CleBGLU18* _F | TCGTGCCGGTTACGACAA | isotig00469 | AT1G52400.3 | β-Glucosidase 18 (BGLU18) |
| *CleBGLU18* _R | CCTGACCGTCCATCTTCACA | | | |
| *ClePBP1* _F | TTGTGGCCTTCGTCTTTGAGT | isotig17838 | AT3G16420.3 | PYK10-binding protein 1 |
| *ClePBP1* _R | AACCCCTTCGGCCTTGAA | | | |
| *CleNAI2* _F | CGACTTCATCAGTAACAGCTCCTAACT | isotig07117 | AT3G15950.1 | DNA topoisomerase-related |
| *CleNAI2* _R | GGAGGAACCTGTGGGAACTCT | | | |
| *CleTSA1* _F | CGCGCTGAAATGCTTAAAGAA | isotig02612 | AT1G52410.2 | TSK-associating protein 1 |
| *CleTSA1* _R | TGAAATGAGAATCCACGATGGTT | | | |
| *CleAGL9* _F | CAGAGCTCTCCGGACACAGTT | isotig04090 | AT1G24260.1 | K-box region and MADS-box transcription factor family protein |
| *CleAGL9* _R | CATGCGTTCCTTACTCTGAAGATC | | | |
| *ClePHOT1* _F | TTTCCGCTCCACCTGAGTTC | isotig12495 | AT3G45780.2 | phototropin 1 |
| *ClePHOT1* _R | TGATACGGAGACGGGAAAGC | | | |
| *CleAGT* _F | CGTTTGGGAAAGGCCACTAG | isotig04516 | AT2G13360.2 | alanine:glyoxylate aminotransferase; It is involved in photorespiration |
| *CleAGT* _R | CTTCTGTGTGCAGTTTTTCAATCC | | | |
| *CleCA1* _F | CTCGCGCGTGTGTCCAT | isotig18828 | AT3G01500.3 | carbonic anhydrase 1 |
| *CleCA1* _R | CGGACCACGAAGGCATCT | | | |
| *CleCER* _F | AGCGGTGATACATCCATTTGC | isotig00800 | AT1G02205.1 | Fatty acid hydroxylase superfamily, associated with production of stem epicuticular wax and pollen fertility. |
| *CleCER* _R | CAGTGGTATCGCGAAGAGCAT | | | |
| *CleER* _F | GCCGTGAAGTGCGAGCAT | isotig12468 | AT2G26330.1 | Leucine-rich receptor-like protein kinase family protein; involved in specification of organs originating from the shoot apical meristem. |
| *CleER* _R | CAACTTATGTGATGGGCACCAT | | | |
| *CleEXT19* _F | GGCAAACAAGGCCAAGACAA | isotig00056 | AT1G26240.1 | Proline-rich extensin-like family protein |
| *CleEXT19* _R | TTCAATCAATATGGCCAATCCTAA | | | |
| *CleATMYB15* _F | CGATGTCCGCACCGATATTT | isotig19642 | AT3G23250.1 | myb domain protein 15 |
| *CleATMYB15* _R | CCGGGAGACATCAGTACTATCGA | | | |
| *ClePSBX* _F | GCTGCCGCGGCTCTAAC | isotig27053 | AT2G06520.1 | photosystem II subunit X |
| *ClePSBX* _R | CAGAGCCAGCGGCTTCA | | | |
| *ClePUB23* _F | CGGAGGGAAGAGCCGATT | isotig08161 | AT2G35930.1 | plant U-box 23; involved in the response to water stress |
| *ClePUB23* _R | TGCACACGACCGCTATGG | | | |
| *CleActin2* _F* | CCACAGCAGAGCGGGAAA | isotig15388 | AT3G18780.2 | actin 2 |
| *CleActin2* _R* | CACAGCGACAAAGGAAAGCTT | | | |

*Used as a reference to quantify the relative gene expression.

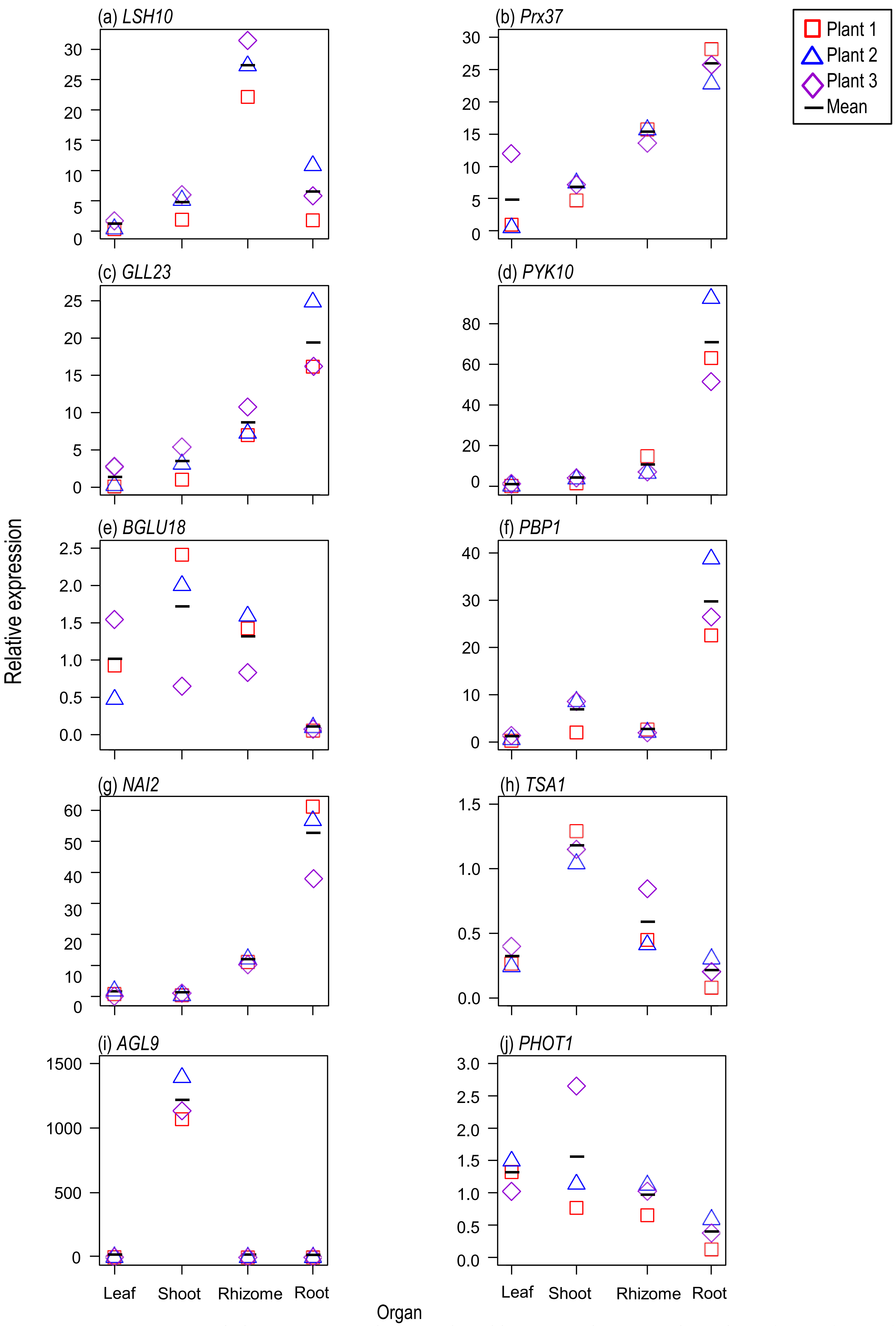


**Supplementary Figure S1.** Relative gene expression quantitated by qPCR of 10 transcripts of *Cardamine leucantha* for four tissues (leaf, shoot, rhizome and root) of three plants (no replicate within plants).

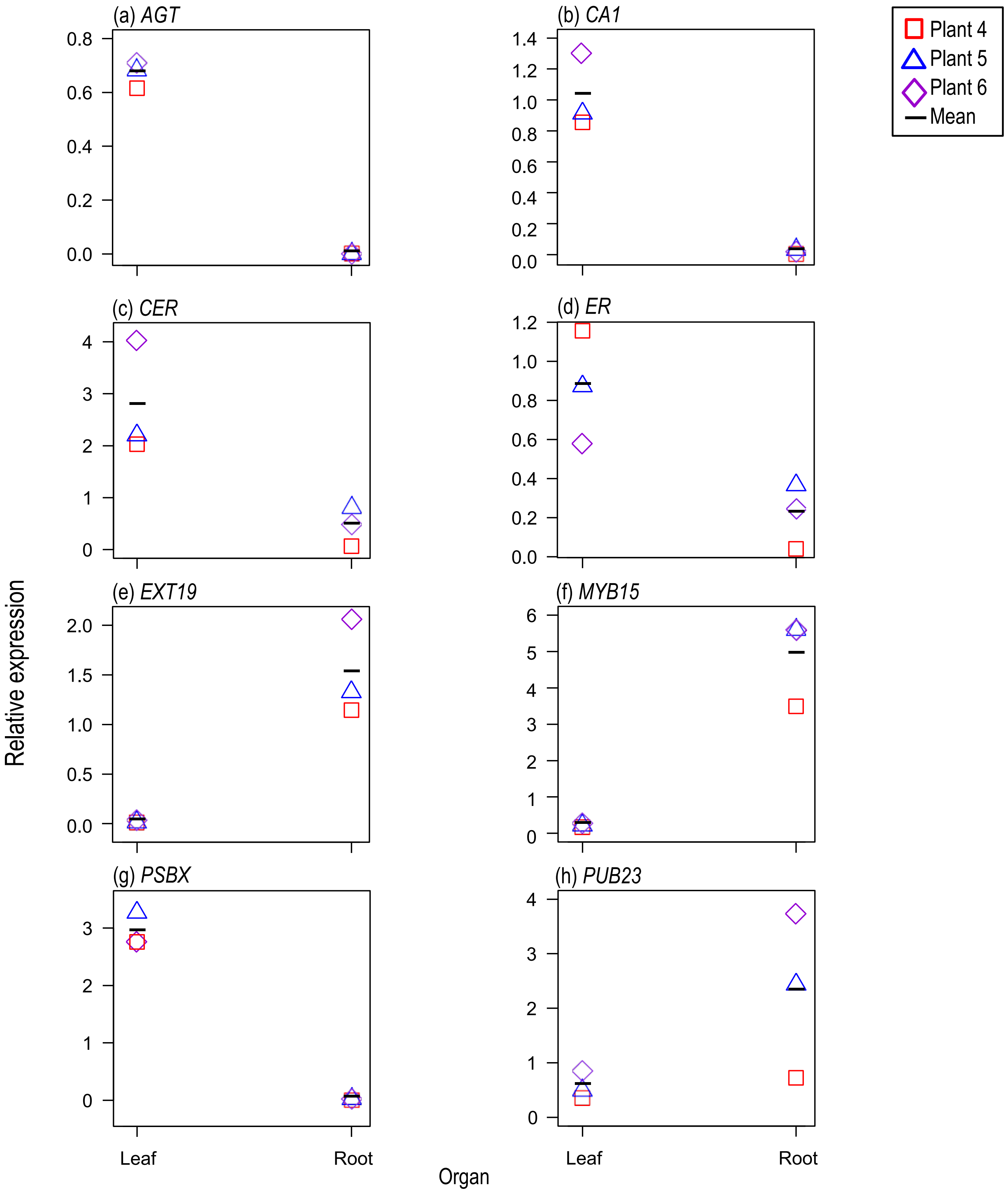


**Supplementary Figure S2.** Relative gene expression quantitated by qPCR of 8 transcripts of *Cardamine leucantha* of above and below ground tissues (leaf and root) of three plants (no replicate within plants).
